# CXR-LLaVA: a multimodal large language model for interpreting chest X-ray images

**DOI:** 10.1007/s00330-024-11339-6

**Published:** 2025-01-15

**Authors:** Seowoo Lee, Jiwon Youn, Hyungjin Kim, Mansu Kim, Soon Ho Yoon

**Affiliations:** 1 Department of Radiology, Seoul National University College of Medicine, Seoul National University Hospital, Seoul, Republic of Korea; 2 AI Graduate School, Gwangju Institute of Science and Technology, Gwangju, Republic of Korea

**Keywords:** Radiography (thoracic), Thorax, Deep learning, Image interpretation, Image interpretation (computer-assisted)

## Abstract

**Objective:**

This study aimed to develop an open-source multimodal large language model (CXR-LLaVA) for interpreting chest X-ray images (CXRs), leveraging recent advances in large language models (LLMs) to potentially replicate the image interpretation skills of human radiologists.

**Materials and methods:**

For training, we collected 592,580 publicly available CXRs, of which 374,881 had labels for certain radiographic abnormalities (Dataset 1) and 217,699 provided free-text radiology reports (Dataset 2). After pre-training a vision transformer with Dataset 1, we integrated it with an LLM influenced by the LLaVA network. Then, the model was fine-tuned, primarily using Dataset 2. The model’s diagnostic performance for major pathological findings was evaluated, along with the acceptability of radiologic reports by human radiologists, to gauge its potential for autonomous reporting.

**Results:**

The model demonstrated impressive performance in test sets, achieving an average F1 score of 0.81 for six major pathological findings in the MIMIC internal test set and 0.56 for six major pathological findings in the external test set. The model’s F1 scores surpassed those of GPT-4-vision and Gemini-Pro-Vision in both test sets. In human radiologist evaluations of the external test set, the model achieved a 72.7% success rate in autonomous reporting, slightly below the 84.0% rate of ground truth reports.

**Conclusion:**

This study highlights the significant potential of multimodal LLMs for CXR interpretation, while also acknowledging the performance limitations. Despite these challenges, we believe that making our model open-source will catalyze further research, expanding its effectiveness and applicability in various clinical contexts.

**Key Points:**

***Question*** *How can a multimodal large language model be adapted to interpret chest X-rays and generate radiologic reports?*

***Findings*** *The developed CXR-LLaVA model effectively detects major pathological findings in chest X-rays and generates radiologic reports with a higher accuracy compared to general-purpose models.*

***Clinical relevance*** *This study demonstrates the potential of multimodal large language models to support radiologists by autonomously generating chest X-ray reports, potentially reducing diagnostic workloads and improving radiologist efficiency.*

## Introduction

Advances in deep learning, marked by the emergence of convolutional neural networks (CNNs) and vision transformers (ViTs), have profoundly impacted radiology [1–3]. Numerous deep learning algorithms have made their way into practical, commercial applications. However, while CNNs and ViTs are adept at specific tasks, such as classification and segmentation, this specialization could limit their ability to address multifaceted challenges in areas such as radiology. Concurrently, the natural language processing domain has witnessed significant breakthroughs, enabling large language models (LLMs), such as ChatGPT, to understand and generate human-like text with remarkable proficiency and unprecedented performance levels in linguistic tasks ranging from text generation to translation [4]. The integration of natural language processing and image processing technologies has led to the development of models that have set new benchmarks in the field, such as contrastive language-image pre-training (CLIP) [5] and the bootstrapping language-image pre-training (BLIP-2) model, which was introduced in 2023 and can interpret the context within images and generate detailed captions [6].

Most LLMs have primarily focused on text processing. However, there is a growing trend towards a multimodal approach involving processing of image, text, and even video data. OpenAI and Google have released general-purpose multimodal models (GPT-4-vision and Gemini-Pro-Vision, respectively). Furthermore, the Large Language and Vision Assistant (LLaVA), an open-source project combining vision encoding with an LLM, has demonstrated exemplary performance across a range of visual tasks [7]. However, it remains unclear how effective these general-purpose models are at interpreting chest X-rays (CXRs). Within the medical domain, there are few specific multimodal models. Google has published results for ELIXR, a model capable of interpreting CXRs, but this model is not publicly available [8]. Similarly, the open-source LLaVA-MED, a model tuned to the medical domain, has been released. However, detailed insights into its proficiency in interpreting CXRs remain limited [9].

Radiologists’ workload has significantly increased over the past three decades, potentially impacting the accuracy of radiologic diagnoses [10]. In response, numerous studies have explored the use of deep learning models to improve diagnostic accuracy and reduce the burden on radiologists [11]. Building on this line of research, our study employed the latest technology, a multimodal LLM, to attempt radiologic report generation for CXRs. This study aimed to develop a multimodal LLM designed for CXR interpretation, while also exploring its potential for autonomous CXR reporting.

A preliminary version of this work has been made publicly available as a preprint on arXiv [12].

## Materials and methods

This retrospective study solely used publicly available datasets and did not require institutional review board approval.

### Data collection

For model training, we included several public CXR datasets, collecting a total of 592,580 frontal CXRs (Table 1) [13–20]. The Medical Information Mart for Intensive Care (MIMIC) dataset provides radiologic reports in a free-text form (Dataset 2, *n* = 217,699), while the other training datasets have multi-class or binary labeling for radiographic abnormalities (Dataset 1, *n* = 374,881). Some datasets contain information regarding lesions’ location, but this information was not utilized.

**Table 1:** Countries of collection, years of publication, and numbers of frontal chest radiographs in the publicly available datasets used for model training and evaluation

Dataset	Country of collection	Year of publication	Numbers of frontal CXRs		
			Training	Validation	Test
Training dataset 1: chest radiograph datasets with pathologic findings labeled					
BrixIA COVID-19 dataset [12]	Italy	2021	3755	470	-
CheXpert train/validation dataset [13]	USA	2019	152,983	19,123	-
NIH dataset [14]	USA	2017	70,671	8833	-
PadChest dataset [15]	Spain	2019	86,438	10,805	-
RSNA COVID-19 AI Detection Challenge [16]	Various countries	2021	5066	634	-
VinDR dataset [17]	Vietnam	2020	14,314	1789	-
Subtotal			333,227	41,654	-
Training dataset 2: chest radiograph dataset with free-text radiologic reports					
MIMIC dataset [18]	USA	2019	193,513	24,186	-
Internal test sets					
MIMIC dataset (randomly selected) [18]	USA	2019	-	-	3000
CheXpert test dataset [13]	USA	2022	-	-	518
Subtotal			-	-	3518
External test set					
Indiana University dataset [19]	USA	2016	-	-	3689

### Adapting a multimodal LLM to CXRs (CXR-LLaVA)

A model influenced by the LLaVA network was developed [7]. LLaVA, which consists of an LLM and an image encoder, converts images into a sequence of image tokens that are then combined with query text tokens for text generation within the LLM. Our primary objective was to fine-tune LLaVA using CXR–radiologic report pairs.

To achieve optimal performance, we developed a custom image encoder from scratch rather than using pretrained weights. We empirically employed the “ViT-L/16” version of the vision transformer as the image encoder. This encoder begins with a convolutional layer that processes 1-channel grayscale CXR images into 1024-dimensional patches. These patches are passed through a series of 24 residual attention blocks, each containing multi-head attention mechanisms and multilayer perceptrons. The output from these blocks is normalized through normalization layers and eventually projected into a higher-dimensional space suitable for multimodal processing. Following the vision encoder, the multimodal projector linearly transforms the 1024-dimensional image tokens into a 4096-dimensional space. These tokens are then integrated into the language model component. In alignment with LLaVA’s framework, we utilized the Large Language Model Meta AI (LLAMA)-2 as our language model [21]. We selected the version with 7 billion parameters due to cost considerations.

The final CXR-LLaVA takes a CXR image and question prompt as input; the image is transformed into image tokens via an image encoder, and the prompt is converted to text tokens through a tokenizer. Both are then fed into a causal language model, which autoregressively generates text responses to the questions. The trained model is available as open-source (https://github.com/ECOFRI/CXR_LLaVA), and its demo can be found at https://radiologist.app/cxr-llava/. Additionally, a comprehensive model card detailing the model’s intended use cases, out-of-scope use, and limitations is provided on the same website to ensure transparency and facilitate further research.

### Training step 1: constructing and training a CXR-specific image encoder

Despite the capabilities of pretrained image encoders in understanding common visual objects, they often fall short in accurately describing radiographic findings. In this section, we propose an image encoder, based on ViT-L/16 and a two-step strategy for training them to learn the radiological context specific to CXR images.

**Fig. 1:**
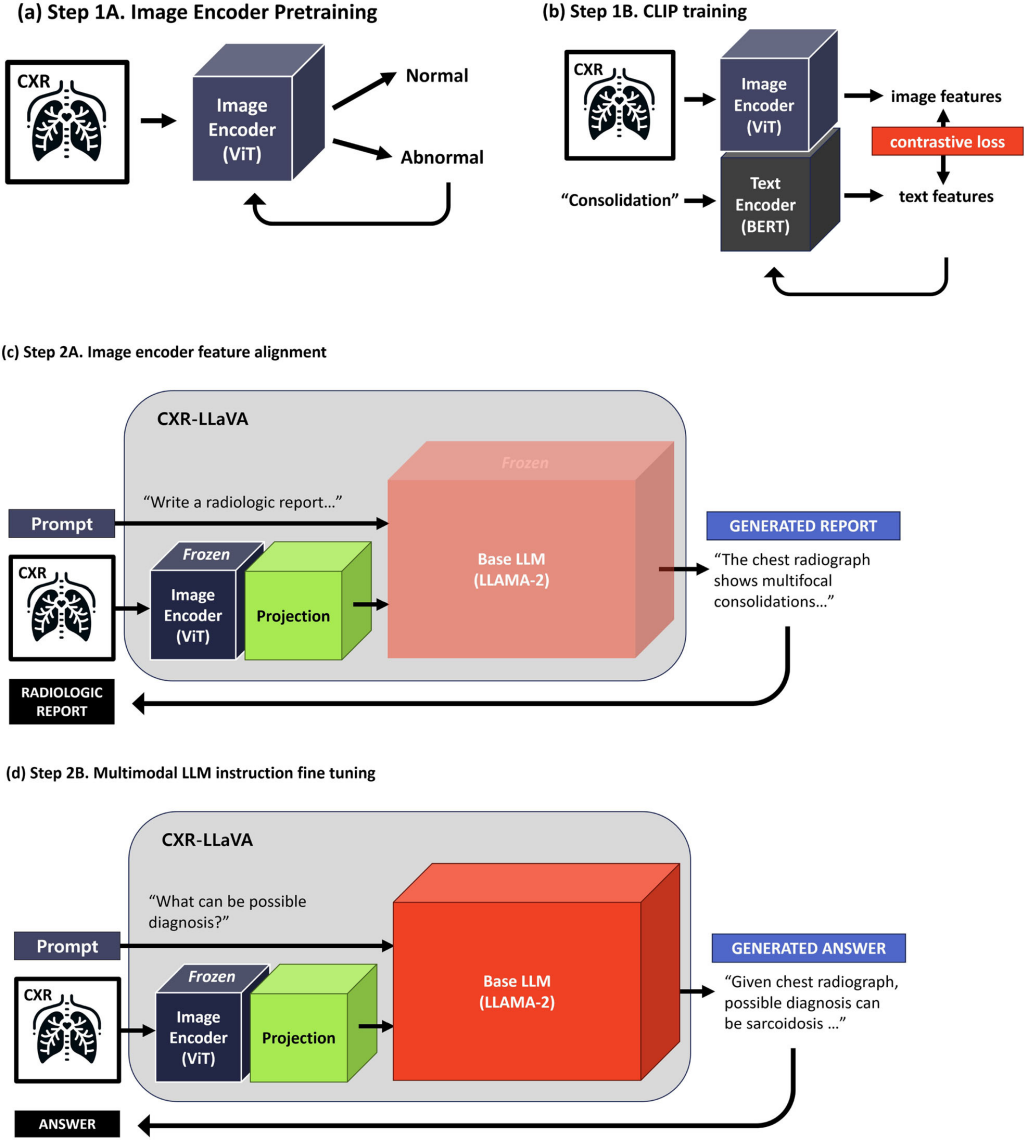
CXR-LLaVA training process. **a** Initially, the image encoder was trained on a basic classification task to differentiate between normal and abnormal CXRs, thereby acquiring fundamental representations of CXRs. **b** Subsequently, the model underwent training with pairs of CXRs and their corresponding pathological findings. This training employed the contrastive language-image pre-training (CLIP) strategy to foster shared representations between images and text. **c** The image encoder was then assimilated into CXR-LLaVA, initiating the alignment of image representations with the large language model (LLM). In this phase, training focused on pairs of CXR images and radiologic reports, with updates confined to the projection layer. **d** Upon successful alignment of the image encoder with the LLM, an instruction fine-tuning process was undertaken. This involved a variety of radiologic reports and question-answer pairs, aiming to refine the model’s capability to interpret CXRs and facilitate more informative interactions. Please note that the figure abstracts from the detailed neural network information, omitting elements such as tokenizer, batch normalization, projection, and linear classification layers

In the first step, a simple classification task was used to train the image encoder (Fig. 1a). The image encoder transformed a CXR image into a representation and then classified an abnormality by adding a simply fully connected layer as a classifier. This classification task enabled the model to learn a fundamental yet crucial ability regarding abnormalities. We used 374,881 image-label pairs from Dataset 1 to train and validate our image encoder. We assigned binary labels: when images had labels associated with pathology, they were labeled as “abnormal,” while those marked as “no finding” were designated “normal.” The detailed implementation and settings are described in the Supplementary material.

In the second step, the image encoder was further trained based on the CLIP strategy to learn complex representations of radiological terms (Fig. 1b) [5]. Using the CLIP strategy, the image encoder learned shared representations between image and text by mapping corresponding image and text pairs closer together and non-corresponding pairs further apart. For instance, an image showing signs of “pleural effusion” would have its corresponding text label vector “pleural effusion” mapped closely to its image vector. This ensures that the model can accurately associate the visual features of pleural effusion in CXRs with the correct textual description, thereby enhancing its ability to correctly identify and describe pleural effusion in new, unseen images. We chose pathological labels provided in the dataset, such as “atelectasis,” “pneumonia,” “pleural effusion” and so on. For images with multiple pathological labels, we connected them using commas. The 592,580 image-text pairs from Datasets 1 and 2 were used in the training and validating process. The performance of the trained image encoder was evaluated and compared with the final model; the detailed process and the performance evaluation are described in the Supplementary material.

### Training step 2: feature alignment and end-to-end fine-tuning of CXR-LLaVA

Before fine-tuning the CXR-LLaVA model, the features from the image encoder, as described in step 1, and language model (i.e., LLaMa-2) were aligned through additional training, where the image encoder and language model weights were frozen, updating only the projection matrix. The aligned image representation was computed by updating the projection matrix using CXR images with refined radiologic reports from Dataset 2 (Fig. 1c).

After aligning the image features, CXR-LLaVA underwent an instruction-tuning process, which was critical for refining the model’s interpretative capabilities (Fig. 1d). This process involved using refined radiology reports and multi-turn question-answer dialogs generated by GPT-4, all based on Dataset 2 (Supplementary materials).

### Internal and external test set composition

For internal model testing, we utilized a randomly selected MIMIC dataset comprising 3000 images and accompanying free-text radiologic reports [19]. These were not used during the model’s training and validation phases. Additionally, we employed the CheXpert test dataset, which consists of 518 images, each binary labeled for 14 findings: atelectasis, cardiomegaly, consolidation, edema, enlarged cardiomediastinum, fracture, lung lesion, lung opacity, no finding, pleural effusion, pleural other, pneumonia, pneumothorax, and support devices [14]. For external model testing, we used a dataset from Indiana University, consisting of 3689 pairs of images and free-text radiologic reports [20].

### Comparison with other multimodal LLMs

To evaluate the performance of our model, we compared its results with those of other publicly available multimodal LLMs, including OpenAI’s GPT-4-vision and Google’s Gemini-Pro-Vision. Despite being in a preview state and not being fine-tuned for CXR report generation, these general-purpose models have shown some potential. For instance, GPT-4-vision has demonstrated a limited ability to detect abnormalities in CXRs and the capacity to solve the United States Medical Licensing Examination tests [22, 23]. However, LLaVA-MED, a model fine-tuned for medical image analysis, failed to generate accurate radiologic reports from CXRs, producing nearly identical reports for diverse CXRs, and was therefore excluded from our study. Other models, such as ELIXR and Med-PALM, which claim the ability to interpret CXRs, were not publicly available and thus were not included in this analysis [8, 24] (Supplementary materials).

### Internal test set evaluation

**Fig. 2:**
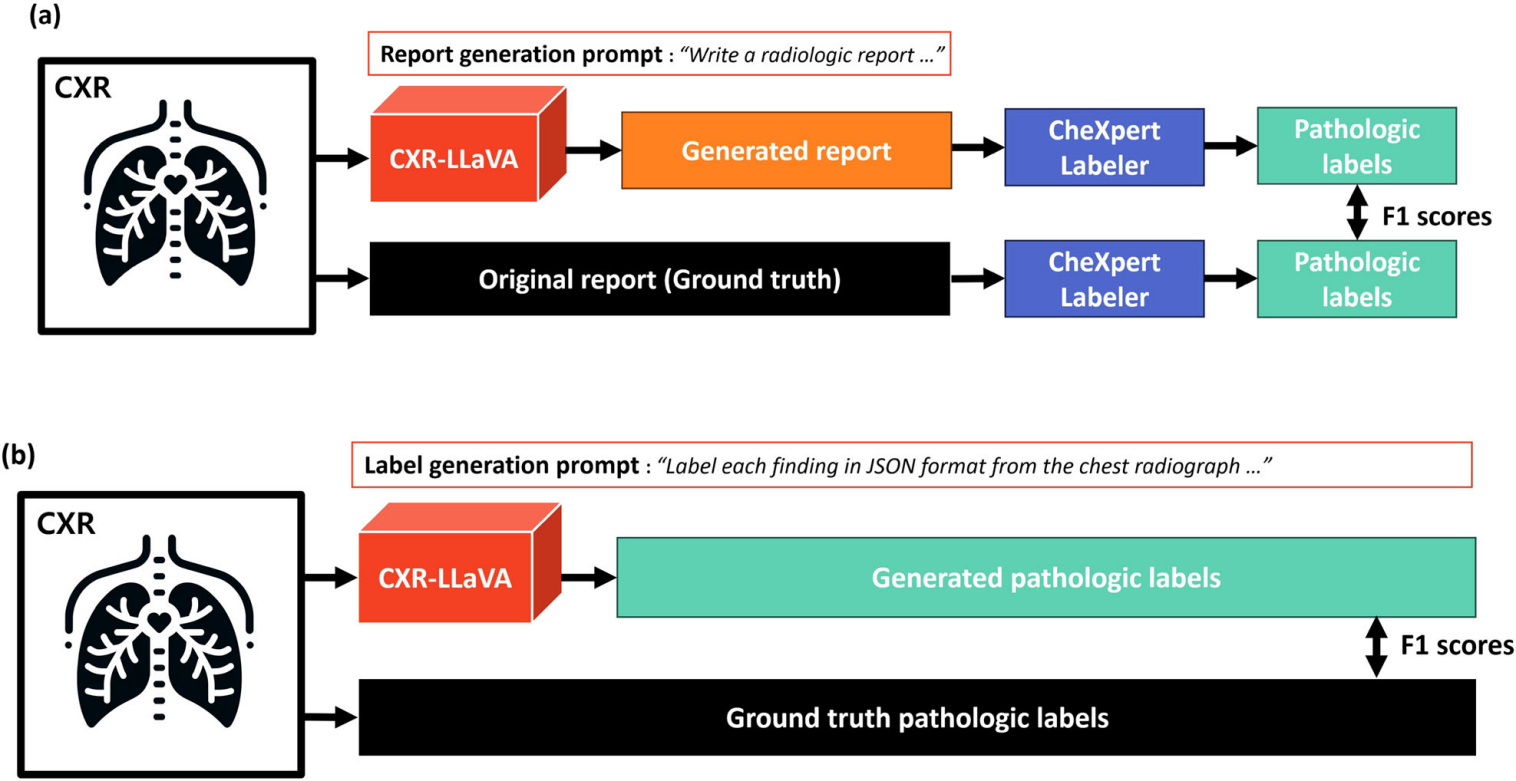
Model evaluation flow diagram. **a** Evaluation of datasets with ground-truth free-text radiologic reports, including the MIMIC internal test set and the Indiana external test set. Pathologic labels were obtained using the CheXpert-Labeler from both the original reports and the model-generated reports, with a subsequent comparison of these results. **b** Evaluation of datasets with established ground-truth pathologic labels, specifically the CheXpert internal test set, involved directly generating pathologic labels from the model using a label generation prompt

To evaluate the performance of radiologic report generation in the MIMIC internal test set, we utilized CheXpert-Labeler to generate pathological labels [14]. This tool analyzes free-text radiologic reports and generates labels such as positive, negative, or uncertain for each pathological finding (atelectasis, cardiomegaly, consolidation, edema, enlarged cardiomediastinum, fracture, lung lesion, lung opacity, no finding, pleural effusion, pleural other, pneumonia, pneumothorax, and support devices). We compared these labels from the model-generated reports with those from the original ground truth reports (Fig. 2a).

For the CheXpert test set, which does not contain ground-truth radiologic reports, we instructed the model to generate binary labels for the same 14 findings. These labels were then compared with the ground truth. This dataset is identical to that used in a previous study where the CheXzero model exhibited expert-level pathology detection capabilities [25]. Therefore, we evaluated our model’s performance against both CheXzero and the average diagnostic performance of three board-certified radiologists, as documented in the same publication (Fig. 2b).

### External test set evaluation and human radiologist evaluation

To evaluate the model’s performance on the Indiana external test set, we employed the same methodology used for the MIMIC internal test set, which involved comparing the labels generated from the model’s reports with the ground truth (Fig. 2a).

To assess the model’s capability for autonomous or semi-autonomous reporting without human radiologist intervention, an evaluation was conducted involving three human radiologists. From the Indiana external test set, 25 abnormal images and 25 normal images were randomly selected. A total of 50 images were used to create 100 report-image pairs, with each image paired with a model-generated report and a ground truth report. The radiologists were presented with these 100 report-image pairs in a random order for evaluation. They rated the acceptability of each report on a 4-point scale: (1) totally acceptable without any revision, (2) acceptable with minor revision, (3) acceptable with major revision, and (4) unacceptable (Supplementary materials).

### Statistical analysis

The model’s performance in generating radiologic reports was assessed using accuracy, sensitivity, specificity, and F1 scores. Cases where the CheXpert-Labeler assigned an “uncertain” label or where the label was not mentioned (missing element) were excluded from our analysis. We included only definite positive or negative labels. Additionally, due to the scarce number of images with labels such as “pleural other” and “fractures,” these were omitted from the analysis. The specific criteria for removing certain labels and the details of the excluded labels are outlined in the accompanying table. To estimate the confidence intervals of the accuracy, sensitivity, specificity, and F1 scores, we utilized non-parametric bootstrapping with 1000 iterations. For the evaluation conducted by human radiologists, the Cochran Q test was employed to determine the statistical significance of differences between the evaluations made by human radiologists and the model.

## Results

### Model performance on the internal test set

Table 2 illustrates the report generation capabilities of our model on the MIMIC internal test set. The model achieved an average F1 score of 0.81, a sensitivity of 0.80, and a specificity of 0.89 for six pathological labels, including cardiomegaly, consolidation, edema, pleural effusion, pneumonia, and pneumothorax. It demonstrated strong performance, with F1 scores exceeding 0.8, in identifying cardiomegaly, edema, and pleural effusion, while its ability to detect pneumothorax was weaker. Overall, the model exhibited higher average F1 scores than GPT-4-vision or Gemini-Pro-Vision.

**Table 2:** Model performance with the MIMIC internal test set

Model performance of each pathologic label in the MIMIC internal test set			
Metric and pathologic label	Models		
	CXR-LLaVA	GPT-4-vision	Gemini-Pro-Vision
Accuracy			
Cardiomegaly	0.79 (0.77, 0.82)	0.65 (0.62, 0.67)	0.65 (0.62, 0.67)
Consolidation	0.93 (0.91, 0.96)	0.80 (0.77, 0.83)	0.55 (0.51, 0.58)
Edema	0.81 (0.77, 0.85)	0.68 (0.61, 0.75)	0.61 (0.58, 0.64)
Pleural effusion	0.87 (0.85, 0.88)	0.66 (0.64, 0.68)	0.53 (0.51, 0.55)
Average for above four pathologies	0.85 (0.84, 0.86)	0.67 (0.65, 0.69)	0.58 (0.57, 0.59)
Pneumonia	0.69 (0.62, 0.76)	0.67 (0.61, 0.74)	0.71 (0.64, 0.77)
Pneumothorax	0.89 (0.88, 0.91)	0.88 (0.87, 0.90)	0.97 (0.91, 1.00)
Overall average	0.86 (0.85, 0.87)	0.73 (0.71, 0.74)	0.59 (0.58, 0.60)
Sensitivity			
Cardiomegaly	0.88 (0.85, 0.90)	0.92 (0.90, 0.93)	0.98 (0.97, 0.99)
Consolidation	0.62 (0.48, 0.75)	0.17 (0.09, 0.27)	0.72 (0.65, 0.80)
Edema	0.85 (0.81, 0.89)	0.69 (0.59, 0.78)	0.80 (0.76, 0.83)
Pleural effusion	0.85 (0.82, 0.87)	0.31 (0.27, 0.35)	0.93 (0.92, 0.95)
Average for above four pathologies	0.85 (0.84, 0.87)	0.62 (0.59, 0.64)	0.91 (0.90, 0.92)
Pneumonia	0.53 (0.42, 0.64)	0.80 (0.73, 0.87)	0.95 (0.91, 0.98)
Pneumothorax	0.35 (0.28, 0.42)	0.02 (0.00, 0.04)	0.00 (0.00, 0.00)
Overall average	0.80 (0.78, 0.82)	0.61 (0.59, 0.64)	0.91 (0.90, 0.93)
Specificity			
Cardiomegaly	0.55 (0.49, 0.61)	0.17 (0.13, 0.20)	0.04 (0.02, 0.06)
Consolidation	0.97 (0.96, 0.99)	0.90 (0.88, 0.93)	0.50 (0.45, 0.54)
Edema	0.75 (0.68, 0.81)	0.67 (0.56, 0.78)	0.39 (0.34, 0.43)
Pleural effusion	0.88 (0.86, 0.90)	0.85 (0.83, 0.87)	0.28 (0.26, 0.31)
Average for above four pathologies	0.85 (0.83, 0.86)	0.71 (0.69, 0.73)	0.29 (0.28, 0.31)
Pneumonia	0.87 (0.78, 0.95)	0.29 (0.16, 0.42)	0.14 (0.06, 0.23)
Pneumothorax	0.97 (0.96, 0.98)	0.99 (0.98, 0.99)	1.00 (1.00, 1.00)
Overall average	0.89 (0.88, 0.90)	0.79 (0.78, 0.80)	0.30 (0.28, 0.31)
F1 score			
Cardiomegaly	0.86 (0.85, 0.88)	0.77 (0.75, 0.79)	0.78 (0.76, 0.80)
Consolidation	0.68 (0.57, 0.78)	0.20 (0.11, 0.29)	0.41 (0.36, 0.47)
Edema	0.84 (0.81, 0.87)	0.71 (0.63, 0.78)	0.69 (0.66, 0.72)
Pleural effusion	0.83 (0.81, 0.85)	0.39 (0.35, 0.43)	0.61 (0.58, 0.63)
Average for above four pathologies	0.84 (0.83, 0.85)	0.62 (0.60, 0.64)	0.67 (0.66, 0.68)
Pneumonia	0.65 (0.54, 0.74)	0.79 (0.73, 0.84)	0.82 (0.77, 0.86)
Pneumothorax	0.46 (0.37, 0.53)	0.03 (0.00, 0.07)	0.00 (0.00, 0.00)
Overall average	0.81 (0.80, 0.82)	0.62 (0.61, 0.64)	0.68 (0.66, 0.69)

In our analysis, specific labels such as “lung lesion,” “lung opacity,” “atelectasis,” “pleural other,” “fracture,” and “support devices” were excluded due to their low frequency, being under 5% in either the negative or positive class or having a sample number below 10, which makes them statistically less significant for a balanced analysis. Additionally, the label “enlarged cardiomediastinum” was not included as it significantly overlaps with “cardiomegaly,” which could lead to redundant data interpretations. For the labels “cardiomegaly,” “consolidation,” “edema,” and “pleural effusion,” we recorded the average score across multiple datasets to facilitate a comprehensive performance comparison of the model on these four critical labels

The model achieved an excellent average F1 score of 0.81, outperforming the GPT-4-vision and Gemini-Pro-Vision models, which scored 0.62 and 0.68, respectively. The model’s sensitivity of 0.80 was higher than GPT-4-Vision’s 0.61 but lower than Gemini-Pro-Vision’s 0.91. Its specificity of 0.89 was higher than both GPT-4-Vision’s 0.79 and Gemini-Pro-Vision’s 0.30

Table 3 presents the model’s pathology detection performance on the CheXpert internal test set [25]. The model achieved an average F1 score of 0.57, a sensitivity of 0.90, and a specificity of 0.67 for five pathological findings: atelectasis, cardiomegaly, consolidation, edema, and pleural effusion. While it performed relatively well in identifying lung opacity, atelectasis, and pleural effusion, its effectiveness in detecting consolidation was lower. This average F1 score of 0.57 is marginally lower than that of CheXzero, which achieved 0.61, and slightly below the 0.62 F1 score reported for human radiologists. No established F1 scores from CheXzero and human radiologists are available for diagnosing lung opacity and support devices, but our model demonstrated commendable F1 scores in detecting these conditions in CXR.

**Table 3:** Model performance with the CheXpert internal test set

Model performance of each pathologic label in the CheXpert internal test set					
Metric and pathologic label	Models				
	CXR-LLaVA	GPT-4-vision	Gemini-Pro-Vision	CheXzero [25]	Human radiologists [25]
Accuracy					
Cardiomegaly	0.66 (0.62, 0.70)	0.31 (0.27, 0.35)	0.48 (0.44, 0.53)	N/A	N/A
Consolidation	0.67 (0.64, 0.71)	0.94 (0.92, 0.96)	0.13 (0.10, 0.16)	N/A	N/A
Edema	0.72 (0.68, 0.76)	0.84 (0.81, 0.87)	0.76 (0.73, 0.80)	N/A	N/A
Pleural effusion	0.77 (0.74, 0.81)	0.58 (0.54, 0.62)	0.78 (0.74, 0.81)	N/A	N/A
Average for above four pathologies	0.71 (0.69, 0.73)	0.67 (0.65, 0.69)	0.54 (0.52, 0.56)	N/A	N/A
Atelectasis	0.77 (0.73, 0.80)	0.70 (0.66, 0.74)	0.69 (0.65, 0.73)	N/A	N/A
Average for above five pathologies	0.72 (0.70, 0.74)	0.67 (0.66, 0.69)	0.57 (0.55, 0.59)	N/A	N/A
Lung opacity	0.82 (0.79, 0.86)	0.68 (0.64, 0.72)	0.54 (0.50, 0.59)	N/A	N/A
Support devices	0.76 (0.72, 0.79)	0.63 (0.58, 0.67)	0.59 (0.55, 0.64)	N/A	N/A
Overall average	0.74 (0.73, 0.75)	0.67 (0.65, 0.68)	0.57 (0.55, 0.59)	N/A	N/A
Sensitivity					
Cardiomegaly	0.90 (0.86, 0.95)	1.00 (1.00, 1.00)	0.57 (0.49, 0.65)	N/A	N/A
Consolidation	0.93 (0.83, 1.00)	0.00 (0.00, 0.00)	0.93 (0.82, 1.00)	N/A	N/A
Edema	0.92 (0.87, 0.98)	0.03 (0.00, 0.06)	0.18 (0.10, 0.27)	N/A	N/A
Pleural effusion	0.96 (0.92, 0.99)	0.70 (0.61, 0.78)	0.02 (0.00, 0.05)	N/A	N/A
Average for above four pathologies	0.93 (0.90, 0.95)	0.63 (0.58, 0.68)	0.36 (0.31, 0.41)	N/A	N/A
Atelectasis	0.85 (0.79, 0.91)	0.00 (0.00, 0.00)	0.02 (0.00, 0.04)	N/A	N/A
Average for above five pathologies	0.90 (0.88, 0.93)	0.44 (0.40, 0.48)	0.25 (0.22, 0.29)	N/A	N/A
Lung opacity	0.90 (0.87, 0.93)	0.73 (0.68, 0.78)	0.94 (0.91, 0.97)	N/A	N/A
Support devices	0.83 (0.79, 0.88)	0.95 (0.92, 0.97)	0.94 (0.90, 0.96)	N/A	N/A
Overall average	0.89 (0.87, 0.90)	0.64 (0.61, 0.67)	0.60 (0.57, 0.63)	N/A	N/A
Specificity					
Cardiomegaly	0.56 (0.51, 0.61)	0.01 (0.00, 0.03)	0.44 (0.39, 0.50)	N/A	N/A
Consolidation	0.66 (0.62, 0.70)	1.00 (1.00, 1.00)	0.09 (0.06, 0.11)	N/A	N/A
Edema	0.68 (0.64, 0.73)	0.99 (0.97, 1.00)	0.87 (0.83, 0.90)	N/A	N/A
Pleural effusion	0.72 (0.68, 0.77)	0.55 (0.51, 0.60)	0.97 (0.95, 0.99)	N/A	N/A
Average for above four pathologies	0.66 (0.64, 0.68)	0.68 (0.66, 0.70)	0.58 (0.55, 0.60)	N/A	N/A
Atelectasis	0.73 (0.68, 0.77)	1.00 (1.00, 1.00)	0.99 (0.98, 1.00)	N/A	N/A
Average for above five pathologies	0.67 (0.65, 0.69)	0.73 (0.71, 0.75)	0.65 (0.63, 0.67)	N/A	N/A
Lung opacity	0.74 (0.68, 0.79)	0.63 (0.57, 0.69)	0.10 (0.06, 0.14)	N/A	N/A
Support devices	0.68 (0.62, 0.74)	0.29 (0.24, 0.35)	0.23 (0.18, 0.29)	N/A	N/A
Overall average	0.68 (0.66, 0.70)	0.68 (0.66, 0.70)	0.56 (0.54, 0.58)	N/A	N/A
F1 score					
Cardiomegaly	0.62 (0.56, 0.67)	0.46 (0.42, 0.51)	0.39 (0.33, 0.45)	0.74 (0.69, 0.79)	0.68 (0.63, 0.72)
Consolidation	0.24 (0.17, 0.31)	0.00 (0.00, 0.00)	0.11 (0.07, 0.15)	0.33 (0.24, 0.42)	0.39 (0.28, 0.49)
Edema	0.50 (0.43, 0.57)	0.05 (0.00, 0.11)	0.19 (0.11, 0.26)	0.60 (0.52, 0.68)	0.58 (0.51, 0.65)
Pleural effusion	0.63 (0.57, 0.69)	0.41 (0.34, 0.47)	0.03 (0.00, 0.09)	0.70 (0.63, 0.76)	0.74 (0.69, 0.78)
Average for above four pathologies	0.53 (0.49, 0.56)	0.40 (0.37, 0.44)	0.21 (0.18, 0.25)	N/A	N/A
Atelectasis	0.69 (0.64, 0.74)	0.00 (0.00, 0.00)	0.04 (0.00, 0.08)	0.65 (0.59, 0.70)	0.69 (0.65, 0.73)
Average for above five pathologies	0.57 (0.54, 0.59)	0.35 (0.32, 0.39)	0.19 (0.17, 0.22)	0.61 (0.57, 0.64)	0.62 (0.59, 0.64)
Lung opacity	0.84 (0.81, 0.87)	0.71 (0.66, 0.75)	0.68 (0.64, 0.72)	N/A	N/A
Support devices	0.78 (0.74, 0.82)	0.72 (0.69, 0.76)	0.70 (0.66, 0.74)	N/A	N/A
Overall average	0.67 (0.65, 0.69)	0.53 (0.51, 0.55)	0.45 (0.43, 0.47)	N/A	N/A

Unbalanced labels like “lung lesion,” “pneumonia,” “pneumothorax,” “pleural other,” and “fracture,” which had a frequency of less than 5% in either the negative or positive class, were not included in the analysis. Additionally, the label “enlarged cardiomediastinum” was not included as it significantly overlaps with “cardiomegaly,” which could lead to redundant data interpretations. For the labels “cardiomegaly,” “consolidation,” “edema,” and “pleural effusion,” we recorded the average score across multiple datasets to facilitate a comprehensive performance comparison of the model on these four critical labels

The model attained an average F1 score of 0.57 for five key pathologies, which was marginally lower than CheXzero’s 0.61 and human radiologists’ 0.62. However, it demonstrated exceptional capability in identifying lung opacity, support devices, and atelectasis. The model’s overall sensitivity was 0.89 and specificity was 0.68, compared to GPT-4-vision’s sensitivity of 0.64 and specificity of 0.68, and Gemini-Pro-Vision’s sensitivity of 0.60 and specificity of 0.56. The sensitivity and specificity for CheXzero and human radiologists were not available, so a direct comparison could not be made

**Fig. 3:**
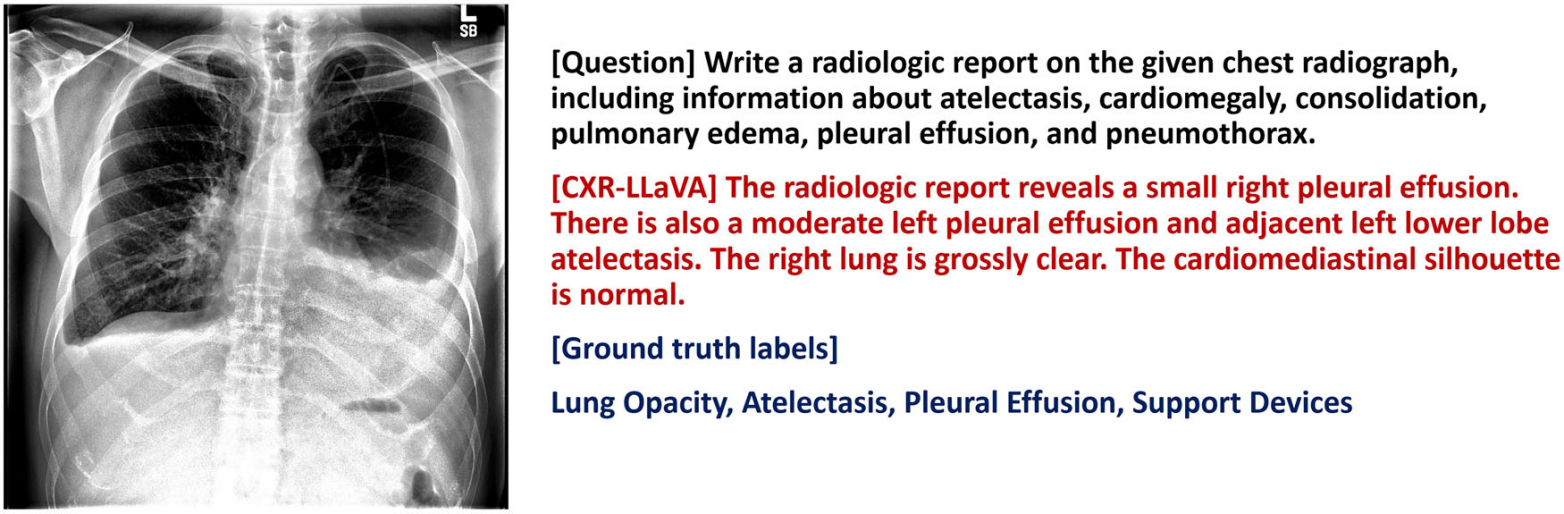
An example of a chest radiograph from the CheXpert internal test set. While the model identified the presence of pleural effusions, atelectasis, and lung opacity, it omitted details about the central catheter (support device)

**Fig. 4:**
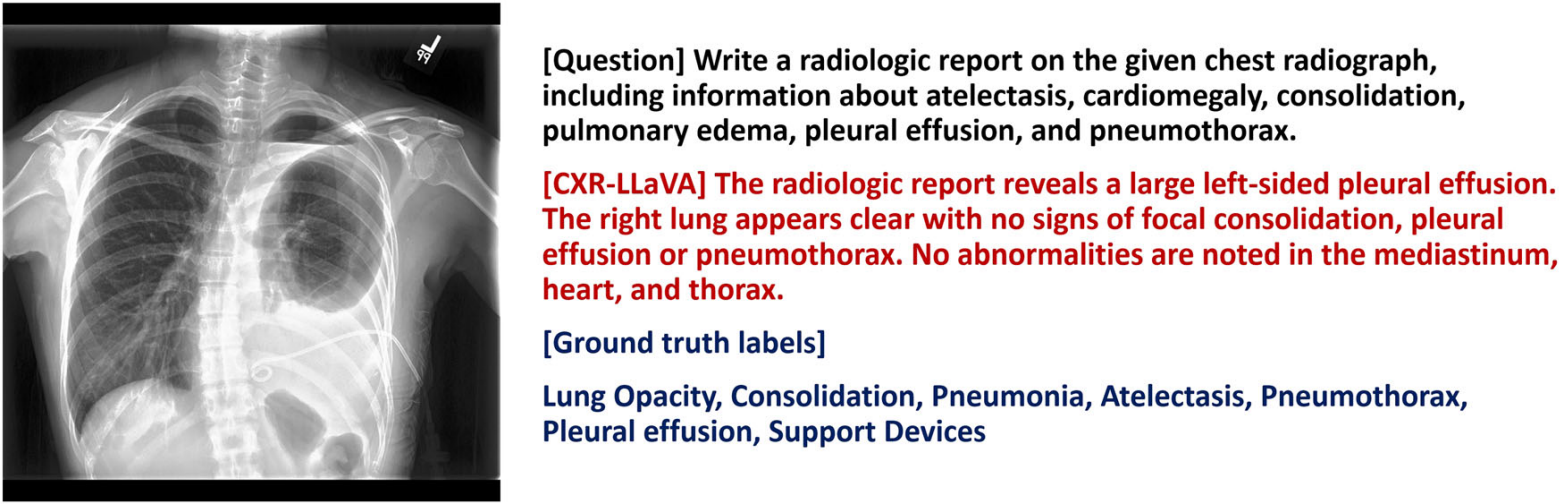
An example of a chest radiograph from the CheXpert internal test set. The model appropriately recognized the left pleural effusion but failed to identify the left pneumothorax and left pleural drainage catheter. The left pneumothorax is a clinically significant finding, indicating that further improvements to the model are necessary

Figure 3 displays an example CXR, highlighting the format of the generated radiologic report. This report effectively pinpoints critical findings, such as bilateral pleural effusion, yet it occasionally overlooks specific details, such as the presence of a central catheter. In contrast, Fig. 4 shows that while the model appropriately recognized and described the left pleural effusion, it failed to describe the left pneumothorax and the left pleural drainage catheter.

### Model performance on the external test set

**Fig. 5:**
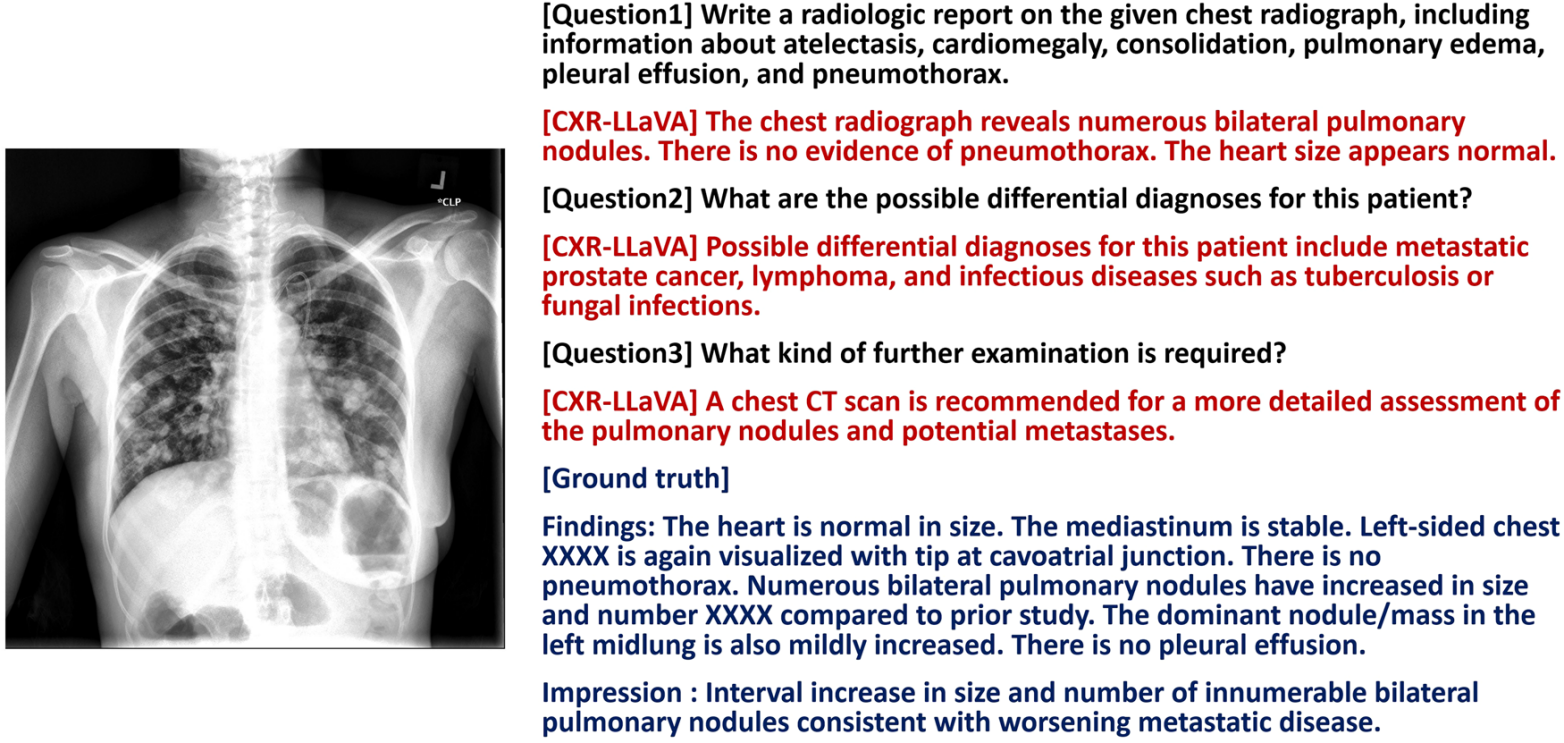
An example of a chest radiograph from the Indiana external test set. The model’s interpretation included information about bilateral pulmonary nodules and suggested a possible diagnosis of lung metastasis or infection, which is reasonable. It also recommended that an additional chest CT scan might be helpful. However, the model could not detect the implanted venous access device

**Fig. 6:**
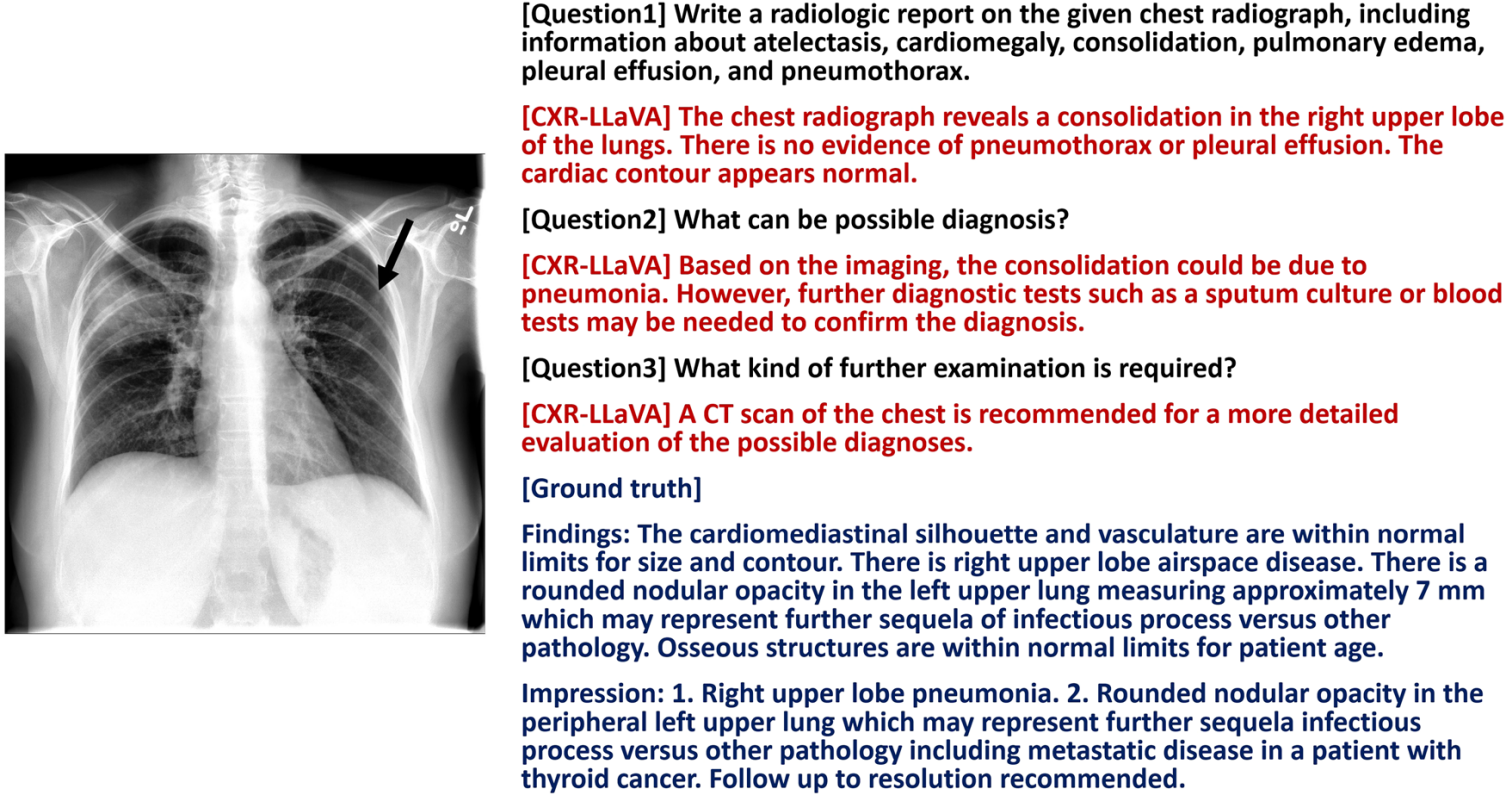
An example of a chest radiograph from the Indiana external test set. The model’s interpretation identified right upper lobe consolidation and proposed pneumonia as a possible diagnosis, which is reasonable. Nonetheless, the model failed to detect a small left upper lung nodule (black arrow)

In the external test set, the model produced an average F1 score of 0.56, a sensitivity of 0.63, and a specificity of 0.93 for detecting cardiomegaly, consolidation, edema, pleural effusion, pneumonia, and pneumothorax. It showed an excellent ability to detect cardiomegaly, edema, and pneumonia, but its performance in detecting pneumothorax was significantly weaker (Table 4). Overall, the model outperformed other models in this regard. A review of several examples showed that the model accurately identified and described the corresponding lesions (Figs. 5 and 6).

**Table 4:** Model performance with the Indiana external test set

Model performance of each pathologic label in the Indiana external test set			
Metric and pathologic label	Models		
	CXR-LLaVA	GPT-4-vision	Gemini-Pro-Vision
Accuracy			
Cardiomegaly	0.72 (0.69, 0.74)	0.35 (0.33, 0.37)	0.41 (0.39, 0.43)
Consolidation	0.93 (0.89, 0.95)	0.93 (0.91, 0.94)	0.74 (0.71, 0.77)
Edema	0.94 (0.90, 0.98)	0.76 (0.63, 0.88)	0.63 (0.57, 0.68)
Pleural effusion	0.94 (0.93, 0.95)	0.88 (0.87, 0.90)	0.66 (0.64, 0.68)
Average for above four pathologies	0.88 (0.87, 0.89)	0.68 (0.66, 0.69)	0.58 (0.56, 0.59)
Pneumonia	0.83 (0.74, 0.90)	0.61 (0.47, 0.76)	0.69 (0.46, 0.92)
Pneumothorax	0.95 (0.94, 0.96)	0.99 (0.98, 0.99)	N/A
Overall average	0.90 (0.89, 0.90)	0.75 (0.74, 0.76)	0.58 (0.56, 0.59)
Sensitivity			
Cardiomegaly	0.67 (0.62, 0.71)	0.81 (0.78, 0.84)	0.81 (0.78, 0.84)
Consolidation	0.33 (0.09, 0.58)	0.08 (0.00, 0.18)	0.18 (0.08, 0.30)
Edema	0.50 (0.25, 0.77)	0.29 (0.00, 0.67)	0.44 (0.29, 0.58)
Pleural effusion	0.71 (0.63, 0.79)	0.19 (0.12, 0.27)	0.71 (0.63, 0.79)
Average for above four pathologies	0.66 (0.62, 0.70)	0.66 (0.63, 0.70)	0.73 (0.70, 0.76)
Pneumonia	0.52 (0.30, 0.71)	0.84 (0.65, 1.00)	1.00 (1.00, 1.00)
Pneumothorax	0.07 (0.00, 0.19)	0.00 (0.00, 0.00)	N/A
Overall average	0.63 (0.59, 0.67)	0.65 (0.61, 0.68)	0.73 (0.70, 0.77)
Specificity			
Cardiomegaly	0.75 (0.71, 0.78)	0.21 (0.19, 0.23)	0.29 (0.27, 0.31)
Consolidation	0.96 (0.93, 0.98)	0.96 (0.95, 0.97)	0.77 (0.74, 0.80)
Edema	1.00 (1.00, 1.00)	0.83 (0.71, 0.95)	0.67 (0.60, 0.72)
Pleural effusion	0.95 (0.95, 0.96)	0.92 (0.90, 0.93)	0.65 (0.64, 0.68)
Average for above four pathologies	0.91 (0.90, 0.92)	0.68 (0.66, 0.69)	0.55 (0.54, 0.57)
Pneumonia	0.95 (0.88, 1.00)	0.47 (0.28, 0.66)	0.00 (0.00, 0.00)
Pneumothorax	0.97 (0.96, 0.98)	1.00 (1.00, 1.00)	N/A
Overall average	0.93 (0.92, 0.94)	0.76 (0.75, 0.77)	0.55 (0.54, 0.57)
F1 score			
Cardiomegaly	0.62 (0.57, 0.65)	0.37 (0.34, 0.39)	0.39 (0.37, 0.42)
Consolidation	0.31 (0.09, 0.50)	0.08 (0.00, 0.17)	0.07 (0.03, 0.11)
Edema	0.67 (0.33, 0.86)	0.25 (0.00, 0.52)	0.28 (0.18, 0.37)
Pleural effusion	0.55 (0.48, 0.62)	0.13 (0.08, 0.18)	0.17 (0.14, 0.20)
Average for above four pathologies	0.59 (0.55, 0.62)	0.33 (0.31, 0.35)	0.30 (0.28, 0.32)
Pneumonia	0.63 (0.42, 0.79)	0.63 (0.45, 0.77)	0.82 (0.56, 0.96)
Pneumothorax	0.05 (0.00, 0.13)	0.00 (0.00, 0.00)	N/A
Overall average	0.56 (0.53, 0.59)	0.33 (0.31, 0.35)	0.30 (0.28, 0.32)

We excluded labels that were unbalanced, with fewer than 10 samples in either the negative or positive category. This included labels such as “lung lesion,” “atelectasis,” “pleural other,” “fracture,” and “support devices.” These were omitted to ensure statistical relevance and balance in the analysis. Additionally, the label “enlarged cardiomediastinum” was not included as it significantly overlaps with “cardiomegaly,” which could lead to redundant data interpretations. Furthermore, “lung opacity” was excluded due to its broad nature, which increases the likelihood of labeling errors by the labeler. For the labels “cardiomegaly,” “consolidation,” “edema,” and “pleural effusion,” we recorded the average score across multiple datasets to facilitate a comprehensive performance comparison of the model on these four critical labels. Gemini-Pro-Vision was excluded from the analysis for “pneumothorax” due to the absence of positive samples, which is indicated as N/A in the table

The model achieved an overall average F1 score of 0.56, excelling particularly in the detection of cardiomegaly (0.62), edema (0.67), and pneumonia (0.63). However, its performance in detecting pneumothorax was notably lower (0.05)

In the evaluation of radiologic report acceptability by human radiologists, the model achieved an “acceptable without any revision” rate of 51.3%, which closely aligns with the 54.0% acceptability rate of ground truth reports. To gauge the model’s capability for autonomous reporting without human radiologist intervention, we defined successful autonomous reporting as reports deemed acceptable either without any revision or with only minor revisions. By this criterion, the model achieved a success rate of 72.7% (Table 5). While this is lower than the 84.0% success rate of ground truth reports, the difference in the rate of autonomous reporting between the model and the ground truth was found to be statistically significant, indicating that the model was somewhat inferior in terms of the autonomous reporting rate. However, the model still maintained a commendable success rate of over 70%.

**Table 5:** Evaluation of radiologic report acceptability by human radiologists from the Indiana external test set

Class	Meaning	CXR-LLaVA	Ground truth	Comparison
A	Acceptable without any revision	77 (51.3%)	81 (54.0%)	
B	Acceptable after minor revision	32 (21.3%)	45 (30.0%)	
C	Acceptable after major revision	8 (5.3%)	6 (4.0%)	
D	Unacceptable	33 (22.0%)	18 (12.0%)	
A + B	Successful autonomous reporting	109 (72.7%)	126 (84.0%)	*p* < 0.001

The model achieved a 51.3% rate (77 cases) of being “acceptable without any revision” (Class A), closely mirroring the 54.0% rate of the ground truth reports. The model’s success rate for autonomous reporting (Class A + B) reached 72.7% (109 cases), slightly lower than the 84.0% for ground truth reports. This difference was statistically significant (*p* < 0.001), highlighting the comparative capabilities and limitations of the model in autonomous radiologic reporting

## Discussion

We successfully developed a multimodal large language model capable of accurately detecting major pathological findings in CXRs and generating free-text radiologic reports. Our model exhibited relatively good performance compared to other publicly available general-purpose multimodal LLMs, such as GPT-4-vision and Gemini-Pro-Vision. We also explored the potential of multimodal LLMs for autonomous or semi-autonomous reporting in chest radiography. However, there are some limitations to our study.

First, the evaluation method we employed has inherent limitations. While we used CheXpert-Labeler to assess the quality of the reports, this tool only evaluates the explicit presence of pathological labels and does not consider the location or number of pathological lesions. As a result, this method may not fully reflect the true accuracy of the generated reports. Second, our model showed poor performance in identifying certain pathological lesions, such as pneumothorax and consolidation. Notably, its diagnostic performance was inferior to that of human radiologists, as shown in the CheXpert internal test set. This might be partly due to the resolution limitations of our model, which processes 512 × 512 pixel images, a lower resolution than the higher-resolution images used by radiologists on specialized monitors. Moreover, our model processes 8-bit images with a grayscale of 256 levels, whereas radiologist monitors can display up to 10 or 12-bit grayscale images, providing finer details. These factors could contribute to the model’s suboptimal performance in detecting subtle lesions. Third, the model intentionally omits descriptions of supporting devices such as feeding tubes and endotracheal tubes in CXRs. During the process of refining original reports with GPT-4 for training data, we removed all mentions of these devices. This decision was due to the varied nomenclature used to describe these devices (e.g., nasogastric tube, Levin tube, Dobhoff tube, feeding tube) and the frequent inclusion of specific numerical details about the tip location. Since language models inherently struggle with processing numerical information accurately, including this varied and numerical information led to hallucinations. Consequently, we excluded it from the training dataset, and the generated reports do not include information about these supporting devices. Fourth, the models to which we compared ours are general purpose and not fine-tuned for CXR interpretation. Therefore, it is not unexpected that our fine-tuned model would outperform them. Nevertheless, it is noteworthy that these general-purpose models still achieved high F1 scores in diagnosing conditions like cardiomegaly and pneumonia. Several non-peer-reviewed public multimodal LLMs, such as Xraygpt, UniXGen, LLM-CXR, and CheXagent have been released for CXR interpretation, but we did not include them in our comparison due to potential dataset overlap, as we utilized a public dataset for both training and testing [26–29]. Fifth, our assessment of the potential of our model for autonomous reporting was based on a limited dataset of just 50 CXRs, which does not mirror real-world clinical settings. Future research should involve larger-scale studies to ensure the safety and efficacy of multimodal LLMs in CXR interpretation. Lastly, as an integral component of CXR-LLaVA is an LLM, hallucinations and confabulations are inherent limitations. The model can generate text that appears plausible but may be incorrect or nonsensical. Therefore, CXR-LLaVA may exhibit hallucinations, and it is unclear under which conditions these hallucinations are most likely to occur or how they can be effectively prevented. Consequently, it is clear that the current model must not be used for clinical purposes without extensive validation.

In conclusion, our study demonstrates the capability of multimodal LLMs to generate radiologic reports that accurately recognize major lesions. By making our model open-source, we aim to promote the development of more capable and accurate models. We are confident that multimodal large language models have considerable potential to assist clinicians, reduce the workload of radiologists in clinical settings, and ultimately improve patient outcomes.

## Supplementary information

ELECTRONIC SUPPLEMENTARY MATERIAL

## Abbreviations

CLIP: Contrastive language-image pre-training
CNN: Convolutional neural network
CXRs: Chest X-ray images
LLM: Large language model
ViT: Vision transformers
