# Supplementary material for: CXR-LLaVA: a multimodal large language model for interpreting chest X-ray images
Source: Eur Radiol. 2025 Jan 15;35(7):4374–86. doi: 10.1007/s00330-024-11339-6 (PMC12166004; doi:10.1007/s00330-024-11339-6)
Supplement: Supplementary file 1 — ELECTRONIC SUPPLEMENTARY MATERIAL [file 330_2024_11339_MOESM1_ESM.pdf]

CXR-LLaVA: a multimodal large language model for interpreting chest X-ray images

ELECTRONIC SUPPLEMENTARY MATERIAL

Detailed Architecture and Training Process of the CXR Image Encoder

We have developed a CXR image encoder utilizing the vision transformer architecture [1]. Empirically, “ViT-L/16” was selected to balance computational cost and performance. Following the training process, this image encoder transforms CXR images (i.e., those with dimensions of 512x512 and single-channel) into a 128-dimensional representation vector, which is subsequently employed for further analysis. Our approach employs a two-staged training strategy to impart radiological context.

In the initial training phase, we first pretrained the image encoder to distinguish between normal and abnormal images. This was accomplished by adding a dense binary classifier to the image encoder. Specifically, we used Dataset 1, and the implementation details are as follows. The model was initialized with random weights. It was trained for up to 100 epochs with a learning rate of 1e-3 using the SGD optimizer and a batch size of 64. Training was conducted for approximately two weeks on a single GPU (NVIDIA A100). The final model was chosen based on the lowest validation loss, and the trained model showed an area under curve of receptor operator characteristics of approximately 0.92 for the binary classification task.

Subsequent to the initial training phase, we incorporated the CLIP (Contrastive Language-Image Pretraining) method to further enhance the image encoder's ability to understand the relationship between text and CXR images [2]. Specifically, we utilized a text encoder based on the Bidirectional Encoder Representations from Transformers (BERT) from prior research [3]. The trained image encoder was then integrated and trained to minimize the contrastive loss between the image vector and text vector. In this step, we trained the encoders twice with different datasets (Dataset 1 and 2). For the initial training stage with Dataset 1, we used pathology labels that lacked location information for learning representation. To accelerate the learning speed, the text encoder was frozen up to step 70k, and then it was unfrozen, and training continued. A learning rate of 1e-2 with an SGD optimizer was used during this phase, and it took about a day using eight NVIDIA A100s. Training was halted when the validation loss reached a plateau. In the subsequent training stage with Dataset 2, radiologic reports, containing not only pathology but also the locations of the pathology, were used to learn the relationship between text and CXR images. The model that demonstrated the lowest validation loss was ultimately selected.

Detailed Architecture and Training Process of the CXR-LLaVA.

We adopted the concept of LLaVA [4] for CXR-LLaVA, which comprises an image encoder trained in the preceding process, a multimodal projection layer, and LLAMA2-7B-CHAT. When a text prompt and a CXR image are input into this model, the LLAMA2 tokenizer initially converts the text prompt into a vector. Simultaneously, the image encoder processes the CXR, producing 128x1024-dimensional image tokens from 512x512 images. These tokens are then transformed into a 5120-dimensional vector through the dense multimodal projection layer. The resulting 5120-dimensional vector is inserted into the specified location within the text prompt. Once it's fed autoregressively into the causal LLM, a response is generated.

The training process for CXR-LLaVA occurred in two stages. In the first stage, all layers except the multimodal projection layer were frozen. Training was then conducted to align the image and text vector spaces. This phase lasted for 1 epoch, using a learning rate of 2e-3 with the Adam optimizer, a batch size of 16, and took about 4 hours on eight NVIDIA A100 40GB GPUs. In the second stage, only the image encoder was frozen, while all other layers were set to be trainable, continuing the training. This stage was conducted over 3 epochs with a learning rate of 2e-5, using the Adam optimizer, a batch size of 16, and took approximately 50 hours on the same GPU setup. No validation was performed during the CXR-LLaVA training process, and the final model iteration was selected as the ultimate model.

The layer composition of CXR-LLaVA is as follows:

```
CXRLLAVAModel(
  (vision_tower): VisionTransformer(
    (conv1): Conv2d(1, 1024, kernel_size=(16, 16), stride=(16, 16), bias=False)
    (patch_dropout): Identity()
    (ln_pre): LayerNorm((1024,), eps=1e-05, elementwise_affine=True)
    (transformer): Transformer(
      (resblocks): ModuleList(
        (0-23): 24 x ResidualAttentionBlock(
          (ln_1): LayerNorm((1024,), eps=1e-05, elementwise_affine=True)
          (attn): MultiheadAttention(
            (out_proj): NonDynamicallyQuantizableLinear(in_features=1024, out_features=1024, bias=True)
          )
          (ls_1): Identity()
          (ln_2): LayerNorm((1024,), eps=1e-05, elementwise_affine=True)
          (mlp): Sequential(
            (c_fc): Linear(in_features=1024, out_features=4096, bias=True)
            (gelu): GELU(approximate='none')
            (c_proj): Linear(in_features=4096, out_features=1024, bias=True)
          )
          (ls_2): Identity()
        )
      )
    )
    (ln_post): LayerNorm((1024,), eps=1e-05, elementwise_affine=True)
  )
  (mm_projector): Linear(in_features=1024, out_features=4096, bias=True)
  (lm_head): Linear(in_features=4096, out_features=32000, bias=False)
  (llama): LlamaModel(
    (embed_tokens): Embedding(32000, 4096)
    (layers): ModuleList(
      (0-31): 32 x LlamaDecoderLayer(
        (self_attn): LlamaSdpaAttention(
          (q_proj): Linear(in_features=4096, out_features=4096, bias=False)
          (k_proj): Linear(in_features=4096, out_features=4096, bias=False)
          (v_proj): Linear(in_features=4096, out_features=4096, bias=False)
          (o_proj): Linear(in_features=4096, out_features=4096, bias=False)
          (rotary_emb): LlamaRotaryEmbedding()
        )
        (mlp): LlamaMLP(
          (gate_proj): Linear(in_features=4096, out_features=11008, bias=False)
          (up_proj): Linear(in_features=4096, out_features=11008, bias=False)
          (down_proj): Linear(in_features=11008, out_features=4096, bias=False)
          (act_fn): SiLU()
        )
        (input_layernorm): LlamaRMSNorm()
        (post_attention_layernorm): LlamaRMSNorm()
      )
    )
    (norm): LlamaRMSNorm()
  )
)
```

Versions of CXR-LLaVA

CXR-LLaVA has two versions, and in this study, we evaluated the model's performance based on version 2.0.1. To provide clarity and facilitate future studies, we outline the key differences between versions 1.0 and 2.0.1 of the CXR-LLaVA model. The primary differences lie in the vision encoder and the base language model used.

Version	Input CXR Resolution	Channels	Vision Encoder	Base LLM
v1.0	512x512	RGB	RN50	LLAMA2-13B-CHAT
v2.0.1 (Latest)	512x512	Grayscale	ViT-L/16	LLAMA2-7B-CHAT

Vision Encoder:

- ✧ Version 1.0 used a CNN-based ResNet50 (RN50) as the vision encoder, processing RGB images.
- ✧ Version 2.0.1 employs a vision transformer-based ViT-L/16, processing grayscale images.

Base LLM:

- ✧ Version 1.0 used the LLAMA2-13B-CHAT model.
- ✧ Version 2.0.1 uses the smaller LLAMA2-7B-CHAT model.

Performance Insights:

- ✧ While direct performance comparisons are challenging due to different training and test datasets, the vision transformer in version 2.0.1 demonstrated better recognition of lesion locations.
- ✧ Version 2.0.1 is more computationally efficient due to the smaller LLM and the use of grayscale images.

CXR-LLaVA Model Card

Intended Use Cases

CXR-LLaVA is designed for generating radiologic reports from chest X-ray images and is intended for research purposes. It can assist researchers in exploring the potential of multimodal large language models in interpreting chest X-rays. The model is suitable for assistant-like chat interactions related to chest X-ray interpretation.

Out-of-Scope Use

- ✧ Use for interpreting non-CXR images or medical imaging modalities not covered in the training data, such as photographs or other types of radiological images, which will result in meaningless outputs.
- ✧ Clinical decision-making or direct patient care.

Training Data

The CXR-LLaVA model was trained on multiple open CXR datasets, including BrixIA, CheXpert, MIMIC, NIH, PadChest, RSNA COVID-19 AI Detection Challenge, and VinDR datasets. Refer to main text for more details.

Model Release

Model (v2.0.1) Release Date: January 14, 2024.
This is a static model trained on an offline dataset.

Ethical Considerations

Research Use Only: The CXR-LLaVA model is intended solely for research purposes. Users must ensure ethical and responsible use within a research setting. It should not be used for clinical diagnosis or treatment without thorough validation and regulatory approval.

Informed Usage

Users must be knowledgeable about the model's capabilities and limitations. They should interpret results within the context of their expertise and be aware of the potential implications of using the model.

Data Privacy

When using the model with patient data, researchers must adhere to all relevant data protection and privacy regulations. Anonymization of patient data is essential to maintain confidentiality and privacy.

Limitations

- ✧ **Domain-Specific Training:** The model was trained exclusively on chest X-ray (CXR) images. Inputting non-CXR images, such as photographs or other types of medical imaging, will result in meaningless outputs.

- **Numerical Data Handling:** The model may struggle with accurately processing numerical data, including specific measurements or quantitative details often found in radiologic reports, such as the exact location or size of abnormalities.
- **Image Quality:** The model processes 512x512 resolution grayscale images. Differences in image resolution or grayscale levels from those used during training could affect the model's performance. Higher resolution images or those with more grayscale levels might provide details that the model cannot accurately interpret.
- **Bias and Generalizability:** The model was trained on specific datasets, which may not fully represent the diversity of clinical cases in different medical settings. This could lead to biases in the model's outputs. Users should interpret results cautiously and consider potential biases.
- **Unpredictable Outputs:** As with all LLMs, the CXR-LLaVA model may produce unpredictable outputs. Safety testing and tuning tailored to specific applications are necessary before deploying any applications involving this model.
- **Regulatory Approval:** The model has not undergone regulatory approval processes, such as FDA clearance. It must not be used for clinical decision-making or direct patient care without such approval.

Important Note

CXR-LLaVA may generate incorrect interpretations of chest X-rays, omit crucial information, or provide inaccurate responses during interactions. Therefore, it should never be used for patient treatment. The model is intended solely for research purposes and should not be relied upon for clinical decision-making or direct patient care.

License Information

CXR LLaVA is available under a Creative Commons NonCommercial License.
Users must obtain the LLAMA-2 license prior to use.

Refining the Radiologic Report and Preparing the LLM Fine-Tuning Dataset

The MIMIC dataset provides free-text radiology reports for CXRs. However, original radiology reports are not suitable for use as LLaVA training data since they contain contextual information that cannot be inferred from a single CXR, like comparisons with previous images and the patient's medical history. We used OpenAI GPT-4 to remove such parts, and the prompt we used is as follows:

> You are skillful radiologist and doing summarization of chest x-ray report.
> Summarize these information from the report.
> Answer to each questions as json format which have "standard report", "conclusion" and "recommendation" as keys.
>
> 1. "standard_report" : Write a standardized radiologic report as one paragraph. Standardized report must include information about abnormality of lungs, mediastinum, heart and thorax.
> 2. "conclusion" : What is the conclusion or impression of the radiologic report? Include only critical information.
> 3. "recommendation" : Should additional radiologic study needed? What type of study should be performed?
>
> Do not include any temporal or time information in standard_report and conclusion. DO NOT USE WORD SUCH AS "new", "previous", "comparison", "stable", "improved", "improving", "decreased", "increased", "changed", "unchanged", "resolved", or "cleared".
> Do not include information about 'comparison with prior study'.
> Do not include information about lateral radiograph.
> Replace any numeric information, such as millimeter or centimeter
> Remove any information about patient age, gender, and medical history.
> Remove any under-bar & blank.
> Remove any information or location about catheter, chest tube, endotracheal tube, PICC, chemoport, central line, nasogastric tube or other medical devices.

In addition to simply generating radiologic report, we also created a Q&A dataset for each chest radiograph to incorporate a question answering feature. The dialogues expanded upon hypothetical question-answer pairs, focusing on details such as the specific locations of anomalies, differential diagnoses, and recommendations for further radiological studies. The instruction-tuning, distinct from the initial training, focused on improving the model's ability to engage in more complex and informative interactions regarding CXRs, beyond simple image interpretation. The fine-tuning encompassed all trainable weights of the model, excluding the image encoder, aiming for a comprehensive enhancement of the model's interpretive and interactive capabilities. Examples are as follows:

> "question1" : Compose a question from the perspective of a student radiologist, inquiring

about the anatomical location, number, or presence of pathology in the chest radiograph.

"answer1" : Write an informative answer to question1.

"question2" : Compose a question that asks possible differential diagnoses from this chest radiograph, without referring to the patient's history.

"answer2" : Write an informative answer to question2.

Comparison with other multimodal LLMs

During the inference process using GPT-4-vision and Gemini-Pro-Vision, we utilized their official application programming interfaces to ensure reliable outcomes. For GPT-4-vision, we used the high-resolution mode and set the model temperature to 0 to increase reproducibility. However, GPT-4-vision often rejected requests to evaluate radiologic images, suggesting that they should be assessed by a healthcare professional, which required us to modify the prompt accordingly. For Gemini-Pro-Vision, we also maintained the model temperature at 0 for consistent reproducibility. The specific prompts utilized during the evaluation process are detailed in the supplementary materials. We used the most recent versions of GPT-4-vision and Gemini-Pro-Vision accessible at that time, specifically from January 1, 2024, to January 3, 2024. For GPT-4-vision, we utilized the 'gpt-4-1106-vision-preview' version. Additionally, to observe how the performance of GPT-4-vision varies with temperature settings, we incrementally increased the temperature from 0 to 1 in steps of 0.2 and monitored the changes in the model's performance.

External test set evaluation and human radiologist evaluation

The readers' rating system was centered around referable abnormalities, defined as findings that necessitate further examination, consultation, or follow-up (e.g., lung masses, nodules, pleural effusion, or pneumothorax). The distinction between minor and major revisions hinged on whether the descriptions adequately covered clinically significant referable abnormalities. A report requiring minor revision might accurately list all referable abnormalities but need slight adjustments in shape, size, or location. Conversely, a report would require major revision if it mentioned several referable abnormalities but only partially described them. A report was considered unacceptable if it failed to mention any referable abnormalities, with potentially serious clinical implications. We defined successful autonomous reporting as reports rated either A) acceptable without any revision or B) acceptable with minor revisions.

Prompts used for evaluating the MIMIC internal test set and Indiana external test set with the GPT-4-vision model.

**System**	We are conducting a study to evaluate image recognition abilities in healthcare. Identify the condition and describe key findings in the image.
**User**	Write a detailed report based on the provided image.
**User**	[CXR image]

Prompts used for evaluating the CheXpert internal test set with the GPT-4-vision model.

**System**	We are conducting a study to evaluate image recognition abilities in healthcare. Identify the condition and describe key findings in the image.
**User**	Label each finding in JSON format. If a finding is present, the value will be 1; if not, the value will be 0. The findings include 'No Finding', 'Enlarged Cardiomediastinum', 'Cardiomegaly', 'Lung Lesion', 'Lung Opacity', 'Edema', 'Consolidation', 'Pneumonia', 'Atelectasis', 'Pneumothorax', 'Pleural Effusion', 'Pleural Other', 'Fracture', and 'Support Devices'.
**User**	[CXR image]

Prompts used for evaluating the MIMIC internal test set and Indiana external test set with the Gemini-Pro-Vision model.

**System**	We are conducting a study to evaluate image recognition abilities in healthcare. Identify the condition and describe key findings in the image.
**User**	Write a radiologic report on the given chest radiograph, including information about atelectasis, cardiomegaly, consolidation, pulmonary edema, and pleural effusion.
**User**	[CXR image]

Prompts used for evaluating the CheXpert internal test set with the Gemini-Pro-Vision model.

**System**	You are a JSON labeler. Try to interpret chest x ray image and answer to the question that user provides.
**User**	Write a radiologic report and label finding of given chest radiograph in JSON formatted string!<br>If a radiologic finding is present, the value will be 1; if not, the value will be 0.<br>EXAMPLE:<br>{<br>"No Finding": 0,<br>"Enlarged Cardiomediastinum": 0,<br>"Cardiomegaly": 0,<br>"Lung Lesion": 0,<br>"Lung Opacity": 0,<br>"Edema": 0,<br>"Consolidation": 0,<br>"Pneumonia": 0,<br>"Atelectasis": 0,<br>"Pneumothorax": 0,<br>"Pleural Effusion": 1,<br>"Pleural Other": 0,<br>"Fracture": 0,<br>"Support Devices": 0<br>}
**User**	[CXR image]

Analysis of GPT-4V Model Performance with Temperature Variation

The performance of the GPT-4V model was evaluated across different temperature settings on the MIMIC internal test set to understand how temperature affects various performance metrics. The temperatures tested ranged from 0 to 1.0 in increments of 0.2. The metrics analyzed include accuracy, sensitivity, specificity, and F1 score. The results are presented below with 95% confidence intervals.

**Temperature**	**Accuracy**	**Sensitivity**	**Specificity**	**F1 Score**
**T=0.0**	0.73 (0.71, 0.74)	0.61 (0.59, 0.64)	0.79 (0.78, 0.80)	0.62 (0.61, 0.64)
**T=0.2**	0.74 (0.72, 0.75)	0.58 (0.56, 0.60)	0.82 (0.81, 0.83)	0.61 (0.59, 0.63)
**T=0.4**	0.73 (0.72, 0.74)	0.58 (0.55, 0.60)	0.81 (0.80, 0.83)	0.60 (0.58, 0.62)
**T=0.6**	0.73 (0.71, 0.74)	0.59 (0.57, 0.62)	0.80 (0.79, 0.81)	0.61 (0.59, 0.63)
**T=0.8**	0.72 (0.70, 0.73)	0.62 (0.60, 0.64)	0.77 (0.76, 0.79)	0.61 (0.59, 0.63)
**T=1.0**	0.70 (0.68, 0.71)	0.64 (0.61, 0.66)	0.73 (0.72, 0.75)	0.61 (0.59, 0.63)

The results indicate that varying the temperature parameter in the GPT-4V model affects different performance metrics. However, the changes in performance metrics are relatively minor. Especially the F1 Score remained stable across all temperatures, suggesting that the model maintains a balance between precision and recall regardless of temperature adjustments. In conclusion, the performance of the GPT-4V model showed minimal variation across different temperature settings, indicating that the temperature parameter does not significantly impact the overall performance of the model.

Diagnostic Performance of the CXR Image Encoder

The CXR image encoder transforms CXR images into vectors. For the diagnostic evaluation on the CheXpert internal test set, the text encoder within CLIP converts the text descriptions "[pathology]" and "no [pathology]" into corresponding text vectors. The distance between the image vector and each text vector is then computed to generate a score. These scores are used to calculate the probability of the presence of pathology using a softmax function. Sensitivity, specificity, F1 scores, and the area under the curve of receiver operating characteristics (AU-ROC) were calculated for the resulting probability scores. The threshold was set to maximize the F1 score, and sensitivity and specificity were calculated based on this optimized threshold, while the AU-ROC was calculated using the continuous probability values.

	CXR Image Encoder				CXR-LLaVA
	**Sensitivity**	**Specificity**	**AU-ROC**	**F1 score**	**F1 score**
**Cardiomegaly**	0.78 (0.71, 0.84)	0.83 (0.79, 0.87)	0.87 (0.84, 0.90)	0.71 (0.66, 0.77)	0.62 (0.56, 0.67)
**Consolidation**	0.28 (0.12, 0.45)	0.89 (0.86, 0.92)	0.56 (0.45, 0.68)	0.18 (0.07, 0.28)	0.24 (0.17, 0.31)
**Edema**	0.65 (0.55, 0.75)	0.84 (0.80, 0.87)	0.80 (0.75, 0.85)	0.51 (0.43, 0.59)	0.50 (0.43, 0.57)
**Pleural Effusion**	0.73 (0.65, 0.81)	0.68 (0.64, 0.73)	0.78 (0.73, 0.82)	0.49 (0.42, 0.55)	0.63 (0.57, 0.69)
**Atelectasis**	0.75 (0.68, 0.81)	0.61 (0.56, 0.66)	0.71 (0.67, 0.75)	0.57 (0.51, 0.62)	0.69 (0.64, 0.74)
**Lung Opacity**	0.90 (0.86, 0.93)	0.50 (0.45, 0.57)	0.80 (0.76, 0.84)	0.76 (0.73, 0.80)	0.84 (0.81, 0.87)
**Support Devices**	0.85 (0.81, 0.90)	0.45 (0.39, 0.51)	0.73 (0.69, 0.77)	0.72 (0.68, 0.76)	0.78 (0.74, 0.82)

When examining the F1 scores, the performance of CXR-LLaVA and the CXR image encoder was generally similar. However, for pleural effusion, atelectasis, and lung opacity, CXR-LLaVA showed higher performance. This suggests that despite the potentially lower performance of the image encoder in generating vector representations, the large language model and projection layer were effectively fine-tuned to distinguish pathological findings in CXR images. This fine-tuning process likely enhanced the ability of the CXR-LLaVA model to identify these specific conditions more accurately than the CXR image encoder.

Label Distribution in the MIMIC Internal Test Set

The MIMIC dataset provides free-text radiology reports. We randomly selected 3,000 reports from this dataset to use as our test set. These reports were then labeled using the CheXpert-Labeler. The distribution of ground truth labels for these 3,000 reports is as follows:

	**Negative**	**Positive**	**Uncertain/Missing**
**Enlarged Cardiomediastinum**	894	238	1868
**Cardiomegaly**	528	950	1522
**Lung Lesion**	20	166	2814
**Lung Opacity**	71	1018	1911
**Edema**	473	541	1986
**Consolidation**	689	226	2085
**Pneumonia**	227	471	2302
**Atelectasis**	29	828	2143
**Pneumothorax**	1665	222	1113
**Pleural Effusion**	1362	859	779
**Pleural Other**	3	62	2935
**Fracture**	42	162	2796
**Support Devices**	46	1268	1686

Label Distribution in the CheXpert Internal Test Set

The CheXpert internal test set provides binary labels for 14 findings. Their distribution is as follows:

	**Negative**	**Positive**
**No Finding**	450	68
**Enlarged Cardiomediastinum**	262	256
**Cardiomegaly**	364	154
**Lung Opacity**	246	272
**Lung Lesion**	509	9
**Edema**	439	79
**Consolidation**	489	29
**Pneumonia**	507	11
**Atelectasis**	360	158
**Pneumothorax**	509	9
**Pleural Effusion**	413	105
**Pleural Other**	518	0
**Fracture**	513	5
**Support Devices**	252	266

Label Distribution in the Indiana External Test Set

The Indiana external test set provides free-text radiology reports. We extracted labels from these 3,689 reports using the CheXpert-Labeler. Their distribution is as follows, and these are considered ground truth labels.

	**Negative**	**Positive**	**Uncertain/Missing**
**Enlarged Cardiomediastinum**	1931	174	1584
**Cardiomegaly**	1823	565	1301
**Lung Lesion**	176	157	3356
**Lung Opacity**	849	605	2235
**Edema**	267	49	3373
**Consolidation**	1098	59	2532
**Pneumonia**	155	82	3452
**Atelectasis**	7	219	3463
**Pneumothorax**	2456	39	1194
**Pleural Effusion**	2595	133	961
**Pleural Other**	5	41	3643
**Fracture**	46	134	3509
**Support Devices**	71	195	3423

References

1 He, K., Zhang, X., Ren, S. & Sun, J. in Proceedings of the IEEE conference on computer vision and pattern recognition. 770-778.

2 Radford, A. et al. in International conference on machine learning. 8748-8763 (PMLR).

3 Boecking, B. et al. in European conference on computer vision. 1-21 (Springer).

4 Liu, H., Li, C., Wu, Q. & Lee, Y. J. Visual instruction tuning. arXiv preprint arXiv:2304.08485 (2023).
